# Dietary acid load and mortality in the Japan Multi-Institutional Collaborative Cohort Study

**DOI:** 10.1038/s41598-025-25081-6

**Published:** 2025-11-21

**Authors:** Taichi Unohara, Takeshi Watanabe, Kokichi Arisawa, Akari Matsuura, Kahori Kita, Yuka Torii, Masashi Ishizu, Sakurako Katsuura-Kamano, Tien Van Nguyen, Jun Otonari, Hiroaki Ikezaki, Keitaro Tanaka, Chisato Shimanoe, Mako Nagayoshi, Yoko Kubo, Takashi Matsunaga, Rieko Okada, Isao Oze, Hidemi Ito, Nobuaki Michihata, Yohko Nakamura, Shiroh Tanoue, Chihaya Koriyama, Sadao Suzuki, Takeshi Nishiyama, Teruhide Koyama, Etsuko Ozaki, Kiyonori Kuriki, Naoyuki Takashima, Keiko Kondo, Takashi Tamura, Keitaro Matsuo

**Affiliations:** 1 Department of Preventive Medicine, Tokushima University Graduate School of Biomedical Sciences, 3-18-15, Kuramoto-cho, Tokushima, 770-8503 Japan; 2 Student Lab, Tokushima University Faculty of Medicine, Tokushima, Japan; 3 Department of Food Nutritional Science, Tokushima Bunri University, Tokushima, Japan; 4 Thai Binh University of Medicine and Pharmacy, Thai Binh, Vietnam; 5 Department of Psychiatric Medicine, Kyushu University Hospital, Fukuoka, Japan; 6 Department of General Internal Medicine, Kyushu University Hospital, Fukuoka, Japan; 7 Department of Preventive Medicine, Faculty of Medicine, Saga University, Saga, Japan; 8 Department of Pharmacy, Saga University Hospital, Saga, Japan; 9 Department of Preventive Medicine, Nagoya University Graduate School of Medicine, Nagoya, Japan; 10 Division of Cancer Epidemiology and Prevention, Aichi Cancer Center Research Institute, Nagoya, Japan; 11 Division of Cancer Information and Control, Aichi Cancer Center Research Institute, Nagoya, Japan; 12 Department of Descriptive Cancer Epidemiology, Nagoya University Graduate School of Medicine, Nagoya, Japan; 13 Cancer Prevention Center, Chiba Cancer Center Research Institute, Chiba, Japan; 14 Department of Epidemiology and Preventive Medicine, Kagoshima University Graduate School of Medical and Dental Sciences, Kagoshima, Japan; 15 Department of Public Health, Nagoya City University Graduate School of Medical Sciences, Nagoya, Japan; 16 Department of Epidemiology for Community Health and Medicine, Kyoto Prefectural University of Medicine, Kyoto, Japan; 17 Laboratory of Public Health, Division of Nutritional Sciences, School of Food and Nutritional Sciences, University of Shizuoka, Shizuoka, Japan; 18 NCD Epidemiology Research Center, Shiga University of Medical Sciences, Otsu, Japan; 19 Department of Cancer Epidemiology, Nagoya University Graduate School of Medicine, Nagoya, Japan

**Keywords:** Dietary acid load, Mortality, Cardiovascular disease, Cerebrovascular disease, Cancer, Cardiovascular diseases, Metabolic disorders, Nutrition, Public health

## Abstract

The impact of diet on the body acid-base balance may be related to the risk of various chronic diseases. This prospective cohort study examined the relationships between the dietary acid load and all-cause and cause-specific mortalities in a large Japanese population. The data of 74,293 participants (aged 35–69 years in the baseline survey) in the Japan Multi-Institutional Collaborative Cohort Study were analyzed. The dietary acid load was estimated using the net endogenous acid production (NEAP) score. Cox proportional hazards regression analyses were performed to estimate hazard ratios (HRs) and 95% confidence intervals (CIs) for all-cause and cause-specific mortalities according to the quartiles of the energy-adjusted NEAP score after adjustments for potential confounders. During a mean follow-up of 11.6 years, 3,755 deaths were identified. Participants with higher NEAP score had increased HR of all-cause (HR 1.18, 95% CI 1.07–1.29) and cause-specific death including cerebrovascular disease death. Sex-stratified analyses showed that NEAP scores were significantly associated with higher HR of all-cause and cause-specific death, including cerebrovascular disease death in male participants, but not in female participants. This study suggests that the dietary acid load may be associated with all-cause and cause-specific mortalities in Japanese male adults.

**Supplementary Information:**

The online version contains supplementary material available at 10.1038/s41598-025-25081-6.

## Introduction

Dietary habits are an important factor contributing to healthy life^1^. A plethora of studies support the relationship between dietary patterns or specific foods and the risk of developing diseases, such as cardiovascular diseases (CVD) and cancer, which are the leading causes of death worldwide^2–4^. Epidemiological studies suggest that the Mediterranean diet exerts protective effects against cardiovascular events and all-cause, CVD, and cancer deaths^5,6^. Furthermore, adherence to the Dietary Approaches to Stop Hypertension diet has been associated with a lower risk of CVD and mortality^7^. Moreover, diet quality or healthy eating patterns assessed by several indices, such as the Alternate Healthy Eating Index, have been linked to a lower risk of CVD and cancer mortalities^8,9^. The relationship between a vegetarian dietary pattern and a lower risk of all-cause and CVD mortalities in male participants has also been reported^10^. Regarding specific foods, the consumption of processed food and red meat has been associated with higher risk of all-cause, CVD, and cancer death^11,12^.

As a potential mechanism for these relationships, the effects of diet on the acid-base balance of the body have been proposed^13^. Diets rich in animal products may induce acidification, while plant-based diets may induce alkalinization^14^. Metabolic acidosis induced by diet may have detrimental effects on health and be one of the causes of various diseases, such as osteoporosis and chronic kidney disease^15,16^. Two commonly used indices to estimate the dietary acid load are the potential renal acid load (PRAL) and net endogenous acid production (NEAP)^17,18^. Both PRAL and NEAP estimate endogenous acid production based on the information of the nutrition from dietary intake. While NEAP is based on the ratio of protein to potassium intake, PRAL is calculated from protein, phosphorus, potassium and calcium intake ^17 18^. These indices correlate with the consumption of acidogenic foods and inversely correlate with the consumption of alkaline foods^19^. A growing body of evidence suggests that diet-induced acidosis is associated with the CVD risk score^20^, type 2 diabetes^21,22^, hypertension^23,24^ and metabolic syndrome (MetS)^25^. These metabolic risk factors may be a cause of diseases such as CVD including cerebrovascular disease or cancer and previous studies reported the relationships between dietary acid load and all-cause and CVD mortalities^26–28^. However, evidence from cause-specific mortality including cerebrovascular disease mortality and sex-stratified analyses remains limited.

Therefore, the present study investigated the relationships between the dietary acid load and all-cause and cause-specific mortalities in a Japanese population, including sex differences.

## Methods

### Study design and participants

The present study was a prospective cohort analysis using data from the Japan Multi-Institutional Collaborative Cohort (J-MICC) study. The J-MICC Study, which is one of the largest cohort studies in Japan, was started in 2005 to identify gene-environment interactions in lifestyle-related diseases, including cancer, in Japanese adults. The Ethics Committees of the Aichi Cancer Center Research Institute (the affiliation of the present principal investigator Keitaro Matsuo) (IRB No. H2210001A), Tokushima University Hospital (IRB No. 466 − 15) and other participating institutions approved the study protocol. Written informed consent was obtained from all participants. The details of the J-MICC Study have already been described^29–31^. The study was performed in accordance with the Declaration of Helsinki.

**Fig. 1:**
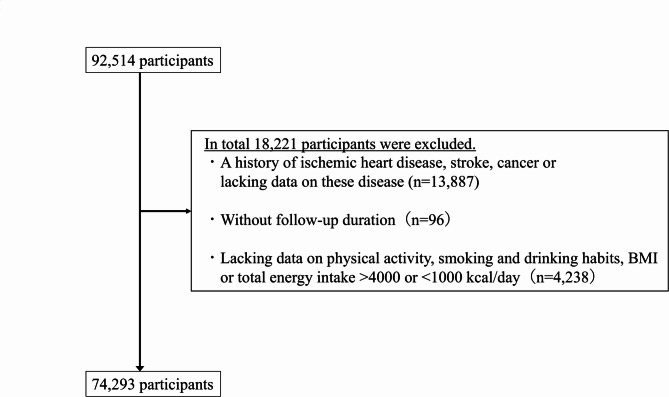
A flowchart for the selection of study participants.

Among 92,514 participants of dataset version 10.16.2023, those with a self-reported history of ischemic heart disease, stroke, or cancer or with missing information on these diseases were excluded (*n* = 13,887). We also excluded participants without follow-up durations (*n* = 96), with missing values for items on smoking and drinking habits or physical activity in the questionnaire or missing data on BMI, or whose total energy intake was extremely high or low (≥ 4,000 or < 1,000 kcal/day) (*n* = 4,238). Therefore, 74,293 participants were included in the present study (Fig. 1).

### Questionnaire

Participants were asked to fill out a self-administered questionnaire that included the following items on medical history, smoking and drinking habits, physical activity, education level, and the frequency of the intake of foods and beverages. Trained staff verified the data obtained.

Physical activity was assessed with a questionnaire based on the short format of the International Physical Activity Questionnaire^32^. The frequency (five categories from none to ≥ 5 times/week) and average duration (six categories from ≤ 30 min to ≥ 4 h) were reported by participants for the following levels of activities: light intensity (e.g., walking and hiking) at 3.4 metabolic equivalent of tasks (METs), moderate intensity (e.g., jogging and dancing) at 7.0 METs, and vigorous intensity (e.g., martial arts and marathon running) at 10 METs. Physical activity was estimated as MET hours/week, which was calculated by multiplying the frequency, average duration, and MET intensity for each level of activity and summing them. Smoking habits were asked as three categories: non-, ex-, and current smokers (≥ once a month). The average number of cigarettes per day and age at the initiation of habitual smoking were also reported. Pack-years were calculated by multiplying the average number of cigarettes per day by the number of years smoked and dividing by 20 (one pack). Drinking habits were also asked as three categories: non-, ex-, and current drinkers (≥ once a month). The frequency and amount of six alcoholic drinks (Japanese sake, shochu, shochu-based cocktails, beer, whiskey, and wine) consumed each time were reported. The ethanol intake (grams per day) of current drinkers was estimated based on the amount of ethanol present in each alcoholic drink. Information on the educational background of participants was asked and classified into the following four categories: ≤9 years, 10–15 years, ≥ 16 years, and unknown.

A validated short food-frequency questionnaire was used to estimate dietary habits^33,34^. The frequency of the consumption of 46 foods and beverages in the previous year was obtained. Regarding the consumption of staple foods, i.e., rice, bread, and noodles, at breakfast, lunch, and dinner, their frequency was categorized into six categories from rarely to every day. Concerning the consumption of the remaining 43 foods and beverages, their frequency was categorized into eight categories from rarely to ≥ 3 times per day. Total energy intake and the intake of 26 nutrients were assessed with the program developed and validated at the Department of Public Health, Nagoya City University School of Medicine^33^. To calculate the intakes of Meat, Vegetables, Fruits and Fish intakes, intakes of chicken, beef and pork, liver and ham, intakes of potato, pumpkin, carrot, broccoli, cabbage, radish, thinly sliced and dried strips of radish, burdock root, mushroom, seaweed, green and yellow vegetables, and light-colored vegetables, intakes of citrus fruits and other fruits, and intakes of large fish, small fish, tuna, shrimp, shellfish, roe, fish cake were summed up respectively.

### Missing data imputation

We used multivariable imputation by chained equations (MICE) to impute missing values in the educational level. Predictors included age, sex, BMI, smoking (pack-years), alcohol intake, research site, physical activity and total energy intake. Ten iterations were performed to stabilize the imputation.

### Dietary acid load estimation

Energy-adjusted NEAP in the present study was calculated using a previously reported method^18,25^. In brief, protein and potassium intakes were energy-adjusted by a residual method and applied to the following formula to calculate NEAP:

$${\text{NEAP (mEq/day) = 54}}{\text{.5}} \times {\text{protein }}\left( {{\text{g}}/{\text{day}}} \right)/{\text{potassium }}\left( {{\text{mEq}}/{\text{day}}} \right) - {\text{1}}0.{\text{2}}$$

### Follow-up and outcome

We followed up participants’ residency and vital status from the baseline survey (February 11, 2004 to March 31, 2014) to the final year of the follow-up, which varied depending on the research sites from the end of 2020 to the end of 2021. The mean duration of the follow-up was 11.6 years and 3,755 deaths (2,463 male and 1,292 female participants) were identified. The breakdown of cause-specific deaths is shown in Supplementary Table 1. The causes of death were defined according to the International Classification of Diseases, 10th Revision (ICD10). The outcome of the present study was mortality from all causes, CVD (ICD10: I00 ~ I99), heart disease (ICD10: I00 ~ I09, I11, I13, I20 ~ I51), cerebrovascular disease (ICD10: I60 ~ I69), and cancer (ICD10: C00 ~ C97). These cause-specific mortality definitions were based on previous studies^26,27^ and reports^35^.

### Statistical analysis

As for the characteristics of participants according to the quartiles of energy-adjusted NEAP scores, the One-way Analysis of Variance (ANOVA) test was applied to continuous variables and the chi-square test to categorical variables. Multivariable Cox proportional hazards regression analyses were performed to examine the relationship between NEAP and mortality. We selected covariates a priori based on previous studies and biological plausibility^25,27,36^. Model 1 was adjusted for fundamental variables such as age (continuous) and sex (two categories); Model 2 was additionally adjusted for the lifestyle factors which may affect the relationship between dietary acid load and mortality such as total energy intake (quartiles), physical activity (quartiles), education level (imputed) (three categories: ≤9 years, 10–15 years, ≥ 16 years), the research site (thirteen categories: Chiba, Aichi Cancer Center, Okazaki, Shizuoka, Daiko, Takashima, Kyoto, Fukuoka, Saga, Kagoshima, Tokushima, the Kyushu Okinawa Population Study, and Shizuoka-Sakuragaoka), and smoking (four categories: never-, 0–20 pack-years, ≥ 20 pack-years, or unknown) and drinking (four categories: never-, ever-, 0–20 g/day, ≥ 20 g/day) habits. Smoking and drinking habits were categorized according to the previous studies^37,38^. BMI (continuous) was also adjusted in Model 3, since body size may have influenced the results. As sensitivity analyses, we also assessed with the model additionally adjusted for antioxidant vitamins, provitamins, and fiber intake taking the influence of vegetables and fruit intake for health other than low dietary acid load into consideration (Model 4). All intakes of energy-adjusted nutrients in Model 4 were handled as continuous variables. Proportional hazards assumptions were tested using the Schoenfeld residuals method and the assumptions were satisfied across all outcomes. Tests for trends were performed by introducing ordinal categorical variables with consecutive numbers 1, 2, 3, and 4 assigned to each quartile group of the NEAP score and using a likelihood ratio test. To assess the differences in the results by research sites, we performed a meta-analysis to combine HRs from each research site. The log-transformed HRs and their standard errors were calculated from 95% CIs. Both fixed-effect and random-effect models (using DerSimonian-Laird method) were used to estimate pooled HRs. Heterogeneity was assessed using the *Q* statistic, *I*^*2*^ and *τ*^*2*^. The significance of the effect modification by sex was examined by including an interaction term generated by multiplying sex and a continuous variable of the NEAP score and using a likelihood ratio test. Stratified analyses by sex were also performed by re-classifying participants into the quartile groups of the NEAP score, physical activity, and total energy intake for males and females separately. To visually estimate the shape of the relationship between dietary acid load and mortality, we performed a restricted cubic spline with 3 knots according to the previous studies^26,27^. As a sensitivity analysis, the analysis with 4 knots was also performed. The median NEAP score of the 1 st quartile group of the NEAP score (35 mEq/day) was selected as the reference. The One-way ANOVA test, chi-square test, and multivariable Cox proportional hazards regression analyses were performed using ANOVA, FREQ, and PHREG and other procedures of SAS version 9.4 (SAS Institute, Inc, Cary, NC, USA). Restricted cubic spline analyses were conducted using the Mkspline and Xblc commands of Stata version 17 (Stata Corp LLC, TX, USA). Meta-analysis was conducted using Python (version 3.11.6) with standard libraries (pandas (version 2.1.1), numpy (version 1.24.4), scipy (version 1.11.3), matplotlib (version 3.8.0)). The imputation of missing variables was performed using statsmodels (version 0.14.0). The level of significance was set at *P* < 0.05.

## Results

Table 1 shows the characteristics of study participants according to the quartile groups of energy-adjusted NEAP scores. Participants with higher NEAP scores were younger, more likely to be male, smoke more cigarettes, drink more alcohol, and less physically active. Moreover, participants with higher NEAP scores had more meat and fish and less vegetables and fruits. Although there was no linear relationship, educational levels also differed according to NEAP scores. Moreover, NEAP scores were significantly different among research sites. The characteristics of study participants according to sex is shown in Supplementary Table 2.

**Table 1:** Characteristics of study participants according to quertiles of energy adjusted net endogenous acid production (NEAP).

	NEAP				
	Q1 (*n* = 18573)	Q2 (*n* = 18573)	Q3 (*n* = 18573)	Q4 (*n* = 18574)	*p*-value
Age (years) ^a^	55.8 (9.0)	54.9 (9.3)	54.5 (9.4)	53.7 (9.6)	< 0.001
Exercise during leisure time (MET-hours/week) ^a^	15.5 (22.6)	14.0 (21.7)	13.3 (21.5)	12.4 (20.7)	< 0.001
Energy intake (kcal/day) ^a^	1705.1 (342.3)	1720.8 (361.1)	1717.7 (350.7)	1701.2 (342.8)	< 0.001
Protein (g/day) ^a^	52.1 (10.3)	52.8 (10.3)	53.3 (10.6)	55.1 (12.0)	< 0.001
Potassium (mg/day) ^a^	2543.2 (481.7)	2161.1 (334.4)	1967.2 (312.9)	1748.1 (302.6)	< 0.001
Energy-adjusted NEAP (mEq/day) ^a^	33.6 (4.3)	41.5 (1.6)	47.1 (1.7)	56.9 (6.3)	< 0.001
Meat intake (g/day) ^a^	33.1 (19.2)	35.9 (19.6)	39.0 (21.5)	45.4 (27.4)	< 0.001
Vegetable intake (g/day) ^a^	237.3 (118.0)	160.8 (74.5)	136.5 (65.6)	111.3 (57.2)	< 0.001
Fruit intake (g/day) ^a^	87.7 (75.1)	60.3 (54.3)	49.0 (45.5)	38.2 (39.0)	< 0.001
Fish intake (g/day) ^a^	46.3 (26.4)	46.8(25.7)	48.7 (27.3)	54.9 (34.2)	< 0.001
BMI ^a^	22.6 (3.2)	22.9 (3.2)	23.0 (3.3)	23.2 (3.4)	< 0.001
Sex ^b^					
Male	6114 (32.9)	7989 (43.0)	8716 (46.9)	9704 (52.2)	< 0.001
Female	12,459 (67.1)	10,584 (57.0)	9857 (53.1)	8870 (47.8)	
Pack-years ^b^					< 0.001
0	12,601 (67.9)	11,311 (60.9)	11,011 (59.3)	10,345 (55.7)	
> 0 and < 20	2302 (12.4)	2689 (14.5)	2874 (15.5)	3044 (16.4)	
> 20	3169 (17.1)	4010 (21.6)	4035 (21.7)	4446 (23.9)	
Unknown	501 (2.7)	563 (3.0)	653 (3.5)	739 (4.0)	
Alchohol drinking ^b^					< 0.001
Never	9149 (49.3)	8022 (43.2)	7692 (41.4)	7026 (37.8)	
Past	408 (2.2)	413 (2.2)	410 (2.2)	516 (2.8)	
> 0 and < 20 g/day	5731 (30.9)	6000 (32.3)	5983 (32.2)	5872 (31.6)	
> 20 g/day	3285 (17.7)	4138 (22.3)	4488 (24.2)	5160 (27.8)	
Education level (years) ^b^					< 0.001
≤ 9	1135 (6.1)	1260 (6.8)	1250 (6.7)	1335 (7.2)	
10 ~ 15	9176 (49.4)	9180 (49.4)	9406 (50.6)	9428 (50.8)	
≥ 16	3509 (18.9)	3745 (20.2)	3766 (20.3)	3884 (20.9)	
Unknown	4753 (25.6)	4388 (23.6)	4151 (22.4)	3927 (21.1)	
Research site ^b^					< 0.001
Chiba	1600 (8.6)	1467 (7.9)	1384 (7.5)	1431 (7.7)	
Aichi Cancer Center	1283 (6.9)	1335 (7.2)	1402 (7.6)	1461 (7.9)	
Okazaki	1480 (8.0)	1507 (8.1)	1443 (7.8)	1409 (7.6)	
Shizuoka	1197 (6.4)	1157 (6.2)	1150 (6.2)	1002 (5.4)	
Daiko	1119 (6.0)	1122 (6.0)	1157 (6.2)	1181 (6.4)	
Takashima	470 (2.5)	488 (2.6)	462 (2.5)	440 (2.4)	
Kyoto	1024 (5.5)	1237 (6.7)	1410 (7.6)	1626 (8.8)	
Fukuoka	2563 (13.8)	2259 (12.2)	2044 (11.0)	1873 (10.1)	
Saga	2470 (13.3)	2662 (14.3)	2783 (15.0)	2804 (15.1)	
Kagoshima	1526 (8.2)	1526 (8.2)	1557 (8.4)	1444 (7.8)	
Tokushima	565 (3.0)	556 (3.0)	536 (2.9)	546 (2.9)	
Kyushu Okinawa Population Study	2140 (11.5)	2056 (11.1)	2020 (10.9)	1984 (10.7)	
Shizuoka-Sakuragaoka	1136 (6.1)	1201 (6.5)	1225 (6.6)	1373 (7.4)	

a) Mean (S.D.)

b) Number (%)

METs: Metabilic equivalent of tasks; NEAP: Net endogenous acid production; BMI: Body mass index

The One-way ANOVA test for continuous variables.

The chi-squared test for categorical variables.

**Fig. 2:**
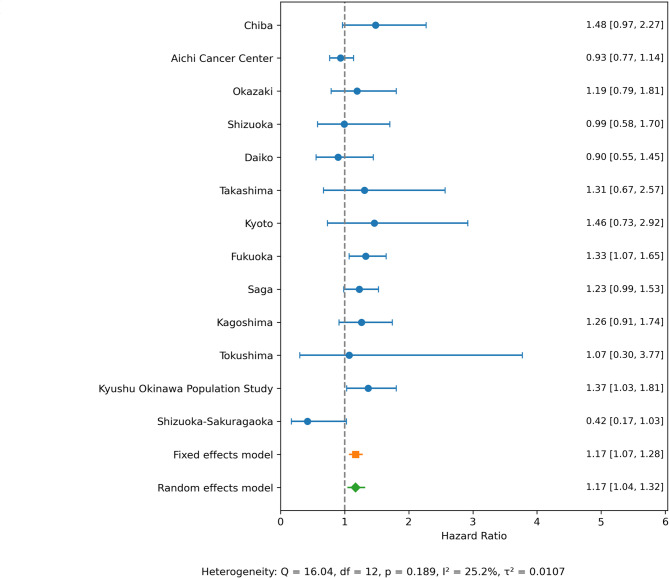
Meta-analysis of HRs and 95% CIs for all-cause mortality associated with NEAP by research site.

**Fig. 3:**
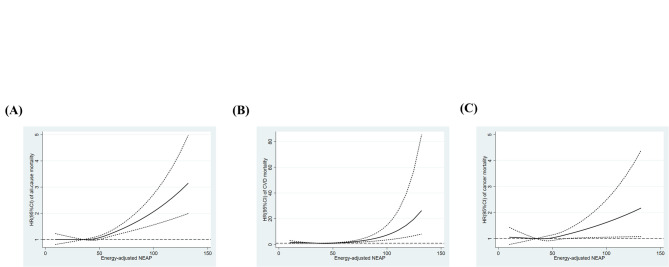
HRs and 95% CIs for all-cause (A), cardiovascular (B), and cancer (C) mortalities associated with NEAP. The model was adjusted for age, sex, total energy intake, physical activity, education level, research site, and smoking and drinking habits. NEAP: Net endogenous acid production; HR: Hazard Ratio; CI: Confidence Interval.

Table 2 shows the relationships between NEAP scores and all-cause and cause-specific mortalities. In Models 1 and 2, a higher NEAP scores were associated with an increased HR for all-cause mortality (Q4, HR for Model 2 1.18, 95% CI 1.07–1.29, *P* for trend < 0.001), CVD (Q4, HR for Model 2 1.50, 95% CI 1.17–1.93, *P* for trend < 0.001), heart disease (Q4, HR for Model 2 1.72, 95% CI 1.19–2.49, *P* for trend < 0.001), and cerebrovascular disease (Q4, HR for Model 2 1.67, 95% CI 1.13–2.47, *P* for trend 0.027). These results were not so much altered by additional adjustments for BMI (Model 3). The relationships between the NEAP score and all-cause mortality (Q4, HR for Model 4 1.14, 95% CI 1.03–1.27, *P* for trend 0.003), and cerebrovascular disease mortality (Q4, HR for Model 4 1.64, 95% CI 1.04–2.56, *P* for trend 0.059) were not markedly affected in the sensitivity analyses with additional adjustments for fiber, carotene, vitamin C, and vitamin E intakes (Table 2). The meta-analysis of the relationships between NEAP scores and all-cause mortality by research site was also performed (Fig. 2). The association between NEAP scores and higher HR of all-cause death was observed both in Fixed effects model (Q4, HR 1.17, 95% CI 1.07–1.28) and Random effects model (Q4, HR 1.17, 95% CI 1.04–1.32). Mean (S.D.) of NEAP scores by research sites are shown in Supplementary Table 3. The results of restricted cubic spline analyses are shown in Fig. 3. A higher NEAP score was associated with higher HR of all-cause (Fig. 3A), CVD (Fig. 3B), and cancer (Fig. 3C) mortalities. The results were not so much altered in the sensitivity analyses with number of knots set to 4 (Supplementary Fig. 1). Sensitivity analyses handling participants who died within one year as censored and excluding participants without the data of education backgrounds are shown in Supplementary Tables 4 and 5, respectively. These results were not so much altered from the results in Table 2.

The results of stratified analyses according to sex are shown in Table 3. A higher NEAP score was associated with increased HR of all-cause and cause-specific mortalities in all models of men, but not in those of women (Table 3). A significant interaction between sex and NEAP scores was observed in CVD mortality (Model 2 *P* for interaction = 0.027, Model 3 *P* for interaction = 0.026, Model 4 *P* for interaction = 0.018) (Table 3).

**Table 2:** HRs and 95% CIs for the association between energy-adjusted NEAP and mortality.

	NEAP				
	Q1	Q2	Q3	Q4	*P* for trend
Participants	18,573	18,573	18,573	18,574	
Person-years	219,671	217,083	214,855	210,707	
All causes					
No. of deaths	850	896	965	1044	
Model 1	1.00	1.04 (0.95–1.14)	**1.13 (1.03–1.24)**	**1.25 (1.14–1.37)**	**< 0.001**
Model 2	1.00	1.02 (0.92–1.12)	1.08 (0.99–1.19)	**1.18 (1.07–1.29)**	**< 0.001**
Model 3	1.00	1.02 (0.93–1.12)	1.09 (0.99–1.19)	**1.18 (1.08–1.30)**	**< 0.001**
Model 4	1.00	1.00 (0.91–1.10)	1.06 (0.96–1.17)	**1.14 (1.03–1.27)**	**0.003**
Cardiovascular disease					
No. of deaths	111	126	135	156	
Model 1	1.00	1.17 (0.90–1.51)	1.27 (0.99–1.64)	**1.55 (1.21–1.98)**	**< 0.001**
Model 2	1.00	1.14 (0.88–1.48)	1.25 (0.97–1.61)	**1.50 (1.17–1.93)**	**< 0.001**
Model 3	1.00	1.14 (0.89–1.48)	1.26 (0.98–1.62)	**1.50 (1.17–1.92)**	**0.001**
Model 4	1.00	1.07 (0.82–1.39)	**1.13 (0.86–1.48)**	1.31 (0.99–1.74)	**0.050**
Heart disease					
No. of deaths	47	59	88	77	
Model 1	1.00	1.26 (0.86–1.85)	**1.89 (1.33–2.71)**	**1.72 (1.19–2.49)**	**< 0.001**
Model 2	1.00	1.24 (0.84–1.82)	**1.89 (1.32–2.71)**	**1.72 (1.19–2.49)**	**< 0.001**
Model 3	1.00	1.24 (0.84–1.82)	**1.90 (1.33–2.72)**	**1.70 (1.17–2.46)**	**< 0.001**
Model 4	1.00	1.09 (0.74–1.62)	**1.57 (1.07–2.30)**	1.32 (0.88–2.00)	0.078
Cerebrovascular disease					
No. of deaths	44	47	34	67	
Model 1	1.00	1.13 (0.75–1.71)	0.85 (0.54–1.33)	**1.78 (1.21–2.62)**	**0.011**
Model 2	1.00	1.10 (0.73–1.66)	0.81 (0.52–1.27)	**1.67 (1.13–2.47)**	**0.027**
Model 3	1.00	1.10 (0.73–1.67)	0.82 (0.52–1.28)	**1.69 (1.14–2.50)**	**0.023**
Model 4	1.00	1.09 (0.71–1.67)	0.80 (0.49–1.29)	**1.64 (1.04–2.56)**	0.059
Cancer					
No. of deaths	505	518	546	607	
Model 1	1.00	1.00 (0.88–1.13)	1.05 (0.93–1.19)	**1.19 (1.06–1.34)**	**0.003**
Model 2	1.00	0.97 (0.86–1.10)	1.00 (0.88–1.13)	1.10 (0.98–1.24)	0.084
Model 3	1.00	0.97 (0.86–1.10)	1.00 (0.89–1.13)	1.10 (0.98–1.25)	0.083
Model 4	1.00	0.96 (0.84–1.09)	0.98 (0.86–1.12)	1.08 (0.94–1.24)	0.204

Model 1: Adjusted for age and sex.

Model 2: Adjusted for variables in Model 1plus total energy intake, physical activity, education level (imputed), research site, and smoking and drinking habits.

Model 3: Adjusted for variables in Model 2 plus BMI

Model 4: Adjusted for variables in Model 2 plus energy-adjusted dietary fiber, carotene, vitamin C and vitamin E.

NEAP: Net endogenous acid production; BMI: Body Mass Index

**Table 3:** HRs and 95% CIs for the association between energy-adjusted NEAP and mortality in male and female participants.

	NEAP						NEAP					
Male	Q1	Q2	Q3	Q4	*P* for trend	Female	Q1	Q2	Q3	Q4	*P* for trend	*P* for Interaction
**Participants**	8130	8131	8131	8131		**Participants**	10,442	10,443	10,442	10,443		
**Person-years**	93,789	93,163	92,595	90,446		**Person-years**	125,006	124,054	122,447	120,817		
**All causes**						**All causes**						
**No. of deaths**	583	608	606	666		**No. of deaths**	355	320	300	317		
**Model 1**	1.00	**1.13 (1.00–1.26)**	**1.14 (1.02–1.28)**	**1.34 (1.20–1.50)**	**< 0.001**	**Model 1**	1.00	0.98 (0.84–1.14)	0.97 (0.83–1.13)	1.14 (0.98–1.33)	0.134	0.163
**Model 2**	1.00	1.11 (0.99–1.25)	1.09 (0.97–1.22)	**1.28 (1.15–1.44)**	**< 0.001**	**Model 2**	1.00	0.96 (0.82–1.12)	0.96 (0.82–1.12)	1.07 (0.92–1.25)	0.462	0.092
**Model 3**	1.00	1.11 (0.99–1.25)	1.10 (0.98–1.23)	**1.29 (1.15–1.44)**	**< 0.001**	**Model 3**	1.00	0.96 (0.82–1.12)	0.95 (0.82–1.11)	1.06 (0.91–1.24)	0.509	0.094
**Model 4**	1.00	1.10 (0.98–1.24)	1.08 (0.95–1.21)	**1.26 (1.11–1.43)**	**0.001**	**Model 4**	1.00	0.92 (0.79–1.08)	0.90 (0.76–1.06)	0.99 (0.83–1.18)	0.877	0.074
**Cardiovascular disease**						**Cardiovascular disease**						
**No. of deaths**	60	80	72	102		**No. of deaths**	59	48	55	52		
**Model 1**	1.00	**1.43 (1.03–2.00)**	**1.31 (0.93–1.85)**	**1.99 (1.44–2.74)**	**< 0.001**	**Model 1**	1.00	0.90 (0.62–1.32)	1.10 (0.76–1.59)	1.19 (0.82–1.73)	0.256	0.052
**Model 2**	1.00	**1.43 (1.02–1.99)** **)**	1.33 (0.94–1.87)	**2.03 (1.47–2.81)**	**< 0.001**	**Model 2**	1.00	0.87 (0.59–1.28)	1.05 (0.73–1.53)	1.07 (0.73–1.56)	0.549	**0.027**
**Model 3**	1.00	**1.43 (1.02–2.00)**	1.34 (0.95–1.89)	**2.01 (1.46–2.78)**	**< 0.001**	**Model 3**	1.00	0.88 (0.60–1.29)	1.06 (0.73–1.53)	1.06 (0.73–1.55)	0.570	**0.026**
**Model 4**	1.00	1.31 (0.93–1.85)	1.18 (0.82–1.69)	**1.73 (1.20–2.48)**	**0.007**	**Model 4**	1.00	0.84 (0.56–1.24)	0.99 (0.66–1.49)	0.99 (0.64–1.53)	0.836	**0.018**
**Heart disease**						**Heart disease**						
**No. of deaths**	28	50	47	56		**No. of deaths**	27	16	25	22		
**Model 1**	1.00	**1.93 (1.22–3.07)**	**1.85 (1.16–2.95)**	**2.36 (1.50–3.72)**	**< 0.001**	**Model 1**	1.00	0.67 (0.36–1.25)	1.12 (0.65–1.92)	1.15 (0.66–2.03)	0.381	0.134
**Model 2**	1.00	**1.92 (1.21–3.04)**	**1.89 (1.18–3.02)**	**2.46 (1.55–3.89)**	**< 0.001**	**Model 2**	1.00	0.65 (0.35–1.20)	1.08 (0.62–1.86)	0.99 (0.56–1.76)	0.678	0.076
**Model 3**	1.00	**1.92(1.21–3.06)**	**1.91 (1.19–3.05)**	**2.41 (1.52–3.81)**	**< 0.001**	**Model 3**	1.00	0.66 (0.35–1.22)	1.08 (0.62–1.86)	0.99 (0.56–1.75)	0.701	0.072
**Model 4**	1.00	**1.72 (1.07–2.76)**	1.62 (0.99–2.66)	**1.99 (1.20–3.30)**	**0.019**	**Model 4**	1.00	0.56 (0.29–1.06)	0.86 (0.47–1.57)	0.73 (0.38–1.42)	0.635	0.053
**Cerebrovascular disease**						**Cerebrovascular disease**						
**No. of deaths**	19	20	19	37		**No. of deaths**	22	26	21	28		
**Model 1**	1.00	1.12 (0.60–2.10)	1.09 (0.58–2.05)	**2.24 (1.29–3.90)**	**0.004**	**Model 1**	1.00	1.28 (0.72–2.26)	1.10 (0.60–2.00)	1.62 (0.93–2.84)	0.148	0.316
**Model 2**	1.00	1.12 (0.60–2.09)	1.07 (0.57–2.03)	**2.25 (1.29–3.95)**	**0.004**	**Model 2**	1.00	1.23 (0.69–2.17)	1.03 (0.56–1.87)	1.44 (0.82–2.54)	0.306	0.246
**Model 3**	1.00	1.12 (0.60–2.10)	1.08 (0.57–2.05)	**2.27 (1.30–3.98)**	**0.004**	**Model 3**	1.00	1.24 (0.70–2.19)	1.04 (0.57–1.89)	1.45 (0.82–2.56)	0.296	0.252
**Model 4**	1.00	1.10 (0.58–2.11)	1.05 (0.54–2.07)	**2.20 (1.16–4.16)**	**0.012**	**Model 4**	1.00	1.23 (0.68–2.22)	1.03 (0.54–1.97)	1.44 (0.75–2.78)	0.379	0.243
**Cancer**						**Cancer**						
**No. of deaths**	361	347	356	391		**No. of deaths**	193	190	166	172		
**Model 1**	1.00	1.03 (0.89–1.20)	1.08 (0.93–1.25)	**1.26 (1.09–1.45)**	**0.002**	**Model 1**	1.00	1.06 (0.86–1.29)	0.97 (0.78–1.19)	1.09 (0.89–1.34)	0.621	0.394
**Model 2**	1.00	1.02 (0.88–1.18)	1.01 (0.88–1.18)	**1.19 (1.03–1.37)**	**0.030**	**Model 2**	1.00	1.03 (0.84–1.25)	0.95 (0.77–1.17)	1.01 (0.82–1.25)	0.917	0.250
**Model 3**	1.00	1.02 (0.88–1.18)	1.02 (0.88–1.18)	**1.19 (1.03–1.38)**	**0.028**	**Model 3**	1.00	1.02 (0.84–1.25)	0.95 (0.77–1.17)	1.00 (0.81–1.24)	0.830	0.248
**Model 4**	1.00	1.01 (0.87–1.18)	1.01 (0.86–1.18)	**1.18 (1.01–1.39)**	0.056	**Model 4**	1.00	0.99 (0.80–1.22)	0.90 (0.72–1.13)	0.95 (0.74–1.21)	0.506	0.218

Model 1: Adjusted for age.

Model 2: Adjusted for age, total energy intake, physical activity, education level(imputed), research site, and smoking and drinking habits.

Model 3: Adjusted for variables in Model 2 plus BMI.

Model 4: Adjusted for variables in Model 2 plus energy-adjusted dietary fiber, carotene, vitamin C and vitamin E.

NEAP: Net endogenous acid production; BMI: Body Mass Index

## Discussion

The present study assessed the relationship between dietary acid load and all-cause and cause-specific mortality. NEAP scores in the present study were correlated with higher meat intake and lower vegetables and fruits intake, and the results may support that the NEAP scores represented diet-induced acidosis (Table 1). The present results showed that higher NEAP scores were associated with an increased HR of all-cause, CVD, heart disease, and cerebrovascular disease mortalities, but not cancer mortality (Models 1 and 2 in Table 2), although the effect size of all-cause mortality was relatively small (Q4, HR for Model 2, 1.18, 95% CI 1.07–1.29). A meta-analysis of the results across research sites was also performed, and not so high heterogeneity was observed (*I*^*2*^ = 25%) (Fig. 2). Moreover, regarding the relationships between NEAP scores and all-cause and cerebrovascular disease mortalities, additional adjustments for dietary fiber, carotene, vitamin C, and vitamin E intakes did not markedly affect the results obtained (Model 4 in Table 2). Restricted cubic spline analyses showed a positive linear relationship between NEAP scores and the hazard of all-cause, CVD, and cancer mortalities (Fig. 3). In addition, a sex-stratified analysis showed that NEAP scores were associated with higher HR of all-cause and cause-specific death in male participants, but not in female participants (Table 3).

A number of epidemiological studies support the relationships between a higher dietary acid load and cardiometabolic risk factors, including hyperglycemia/diabetes, elevated triglycerides, obesity, hypertension, and MetS^19,21–25,39,40^. These relationships are plausible because a number of mechanisms have been proposed, such as increased cortisol production and subsequent insulin resistance caused by diet-induced metabolic acidosis^41,42^.

On the other hand, evidence for the relationships between the dietary acid load and all-cause and cause-specific mortalities is still limited^26–28^. To the best of our knowledge, three previous studies investigated this issue^26–28^ and showed that a higher dietary acid load was associated with an increased risk of all-cause and CVD mortalities^26–28^. The present results are consistent with these findings (Table 2). These findings are reasonable because cardiometabolic risk factors, which may be exacerbated by higher dietary acid load, are closely related to CVD events and mortality^43,44^. Moreover, the present results were consistent with findings from previous studies showing no association between a higher dietary acid load and higher HR of cancer death (Table 2). However, NEAP scores were associated with higher HR of cancer death in male participants in the present study (Q4, HR for Model 2, 1.19, 95% CI 1.03–1.37, HR for Model 3, 1.19, 95% CI 1.03–1.38, HR for Model 4, 1.18(1.01–1.39) (Table 3), although the effect size was smaller than CVD death. We previously showed that a number of cardiometabolic risk factors, such as metabolic phenotypes and MetS, were associated with higher HR of cancer^38,45^. Therefore, the dietary acid load may play some roles not only in CVD mortality, but also cancer mortality.

Previous studies conducted in Sweden and Iran suggested that excess diet acidity and alkalinity were both linked to an increased risk of death^26,28^, while a study conducted in Japan showed that only acidity appeared to increase the risk of death^27^. Restricted cubic spline analyses in the present study revealed a relationship between a higher dietary acid load and mortality (Fig. 2). The reason for this inconsistency remains unclear; however, a number of reasons have been discussed, such as differences in the levels of the dietary acid load score^28^ and differences in culture and food sources between Japan and other countries^27^.

In the present study, participants with higher NEAP scores were at an increased hazard of cerebrovascular mortality (Table 2). Compared to the participants in Q1 group of NEAP scores, participants in Q4 group have 67% increased hazard of cerebrovascular disease death (Model 2, Table 2). In a previous prospective cohort study conducted in Japan, although there was a tendency for a positive association between dietary acid load and higher HR of cerebrovascular disease death, it was not significant^27^. Therefore, this may be the first study to show a relationship between the dietary acid load and cerebrovascular disease mortality. Cerebrovascular disease is roughly categorized into hemorrhagic stroke (e.g., intracerebral and subarachnoid hemorrhage) and non-hemorrhagic (ischemic) stroke^46,47^. In this study, hemorrhagic stroke was the main cause of cerebrovascular death (*n* = 128 in 192 participants with cerebrovascular death, Supplementary Table 1). While the incidence of hemorrhagic stroke is generally higher in Eastern Asia than in Western populations, the number of hemorrhagic stroke cases is smaller than that of non-hemorrhagic stroke cases in Eastern Asia^47^. However, hemorrhagic stroke is generally fatal^48,49^, and in a previous study that included cohorts from China and Japan, 67% of fatal strokes were hemorrhagic^47^. Therefore, the high percentage of hemorrhagic stroke deaths in the present study appears to be reasonable. Among cardiometabolic risk factors, hypertension may be a major risk factor for both types of hemorrhagic stroke: intracerebral and subarachnoid hemorrhage^47,50,51^. Although various cardiometabolic risk factors may be exacerbated by diet-induced metabolic acidosis, the most well-established relationship is between the dietary acid load and hypertension^19,23,24^. Moreover, this is corroborated by evidence showing that nutritional intake, such as potassium, which inversely correlates with the dietary acid load, may exert preventive effects against hypertension^52^. The impact of the dietary acid load on hypertension may account for the markedly increased hazard of death from cerebrovascular disease, many cases of which were hemorrhagic in the present study.

Sex differences in the relationship between the dietary acid load and the hazard of death may also be an important finding of the present study, with a significant interaction between sex and dietary acid load on the hazard of CVD mortality (Model 2 *P* for interaction 0.027, Model 3 *P* for interaction 0.026, Model 4 *P* for interaction 0.018) (Table 3). It may be attributed to the lower acidic state in females than in males (mean energy-adjusted NEAP score ± S.D. 46.4 ± 9.2 in females and 43.5 ± 9.3 in males). Moreover, renal function, which plays an important role in adjusting the acid-base balance, may differ between males and females^53^. A study in Japan showed that female participants appeared to be protected from developing end-stage renal disease^54^. Furthermore, sex hormones may make differences in cardiometabolic risk factors such as insulin resistance^55^, hypertension^56^, and dyslipidemia^57^. The modulation by sex hormones of cardiometabolic risk factors due to high dietary acid load may be a cause of the differences in the association between NEAP scores and high HR of CVD death in the present study. On the other hand, differences in diet reporting between sex or residual confounding may also affect the results.

High alkaline foods, such as vegetables and fruits, reduce the risk of metabolic acidosis, and are also abundant in nutrients that may exert preventive effects against CVD and cancer, such as antioxidant vitamins and fiber^36^. However, in the model adjusted for dietary fiber, carotene, vitamin C, and vitamin E intakes in the present study, the relationship between a higher dietary acid load and an increased hazard of mortality in male participants was retained (Table 3). These results suggest that the relationship between a higher dietary acid load and increased mortality may not be completely attributed to a low intake of dietary fiber and vitamins present in high alkaline foods.

The strength of the present study is the large number of study participants in a relatively new cohort, and, thus, the data obtained may reflect contemporary dietary habits in Japan. The large sample size enabled us to adjust for various important potential confounders. The relatively low heterogeneity in the meta-analysis of results across research sites supports the generalizability of the results of the present study in Japan. However, this study also has some limitations. First, only NEAP was used as an index of the dietary acid load because data on magnesium and phosphorus intakes were unavailable for the calculation of PRAL scores. This may lead to underestimation of the net al.kaline contribution of plant-based foods and limit comparability with studies that have used PRAL, although a previous study reported a strong correlation between NEAP and PRAL scores in a Japanese population (Spearman’s correlation coefficient = 0.97)^27^. Second, since NEAP scores were calculated from dietary information obtained through self-reported questionnaires, a misclassification may have been introduced. Furthermore, the information on dietary intake was only at baseline and we could not assess the change of diet over time. This may also be a cause of misclassification of NEAP scores. However, these misclassifications of NEAP scores were likely to be non-differential, and the effect may be towards the null result. Moreover, a misclassification in the causes of death may have occurred. Although the causes of death were defined according to ICD10, for patients who had multiple probable causes of death, only one underlying cause of death was selected by doctors. The misclassification of the causes of death may result in the HR of cause-specific mortality being similar to the HR of all-cause mortality. The difference of the final date of follow-up across research sites may also introduce bias. Reverse causality, where a pre-existing health condition influences dietary habits, should also be mentioned. However, this study attempted to mitigate the influence of reverse causality by excluding participants with diseases at baseline, or by conducting a sensitivity analysis handling early death cases as censored. Furthermore, as is the case for all observational studies, it was not possible to completely rule out the effect of residual confounding factors including environmental exposure or air pollution; however, various potential confounding factors were adjusted for. In addition, it may be difficult to directly apply the results obtained herein to other populations in different countries because this study was conducted on a Japanese population. For example, Japanese dietary pattern which is rich in fish intake (positively correlated with NEAP scores in the present study (Table 1, Supplementary Table 2)) may affect the influence of dietary acid load on mortality^58^.

In conclusion, the present study suggests that a high dietary acid load may be associated with all-cause and cause-specific mortalities, including cerebrovascular disease mortality, in Japanese adults. Furthermore, sex-stratified analyses suggested that these relationships may be specific to male adults. Further research is needed to investigate the relationship between the acid-base balance and mortality, as well as the underlying mechanisms.

## Supplementary Information

Below is the link to the electronic supplementary material.

Supplementary Material 1

Supplementary Material 2

Supplementary Material 3

## Data Availability

The data needed to replicate the results of the study are available upon reasonable request to the corresponding author and after approval by all the participating institutions.

## Abbreviations

CVD: Cardiovascular disease
PRAL: Potential renal acid load
NEAP: Net endogenous acid production
J-MICC: Japan Multi-Institutional Collaborative Cohort
METs: Metabolic equivalent of tasks
ICD-10: International Classification of Diseases, 10th Revision
MetS: Metabolic Syndrome
HR: Hazard Ratio
CI: Confidence Interval
ANOVA: Analysis of Variance

## References

[1] Hooper, L. Primary prevention of CVD: diet and weight loss. *BMJ Clin Evid* **2007** (2007). PMC2943801 19450364

[2] Thomas, H. et al. Global atlas of cardiovascular disease 2000–2016: the path to prevention and control. *Glob Heart*. **13**, 143–163. 10.1016/j.gheart.2018.09.511 (2018). 30301680 10.1016/j.gheart.2018.09.511

[3] Nagai, H. & Kim, Y. H. Cancer prevention from the perspective of global cancer burden patterns. *J. Thorac. Dis.* **9**, 448–451. 10.21037/jtd.2017.02.75 (2017). 28449441 10.21037/jtd.2017.02.75 PMC5394024

[4] Nestel, P. J. & Mori, T. A. Dietary patterns, dietary nutrients and cardiovascular disease. *Rev. Cardiovasc. Med.* **23**, 17. 10.31083/j.rcm2301017 (2022). 35092209 10.31083/j.rcm2301017

[5] Rosato, V. et al. Mediterranean diet and cardiovascular disease: a systematic review and meta-analysis of observational studies. *Eur. J. Nutr.* **58**, 173–191. 10.1007/s00394-017-1582-0 (2019). 29177567 10.1007/s00394-017-1582-0

[6] Mitrou, P. N. et al. Mediterranean dietary pattern and prediction of all-cause mortality in a US population: results from the NIH-AARP diet and health study. *Arch. Intern. Med.* **167**, 2461–2468. 10.1001/archinte.167.22.2461 (2007). 18071168 10.1001/archinte.167.22.2461

[7] Jones, N. R. V., Forouhi, N. G., Khaw, K. T., Wareham, N. J. & Monsivais, P. Accordance to the dietary approaches to stop hypertension diet pattern and cardiovascular disease in a British, population-based cohort. *Eur. J. Epidemiol.* **33**, 235–244. 10.1007/s10654-017-0354-8 (2018). 29318403 10.1007/s10654-017-0354-8 PMC5871645

[8] Schwingshackl, L., Bogensberger, B. & Hoffmann, G. Diet quality as assessed by the healthy eating Index, alternate healthy eating Index, dietary approaches to stop hypertension Score, and health outcomes: an updated systematic review and Meta-Analysis of cohort studies. *J. Acad. Nutr. Diet.* **118**, 74–100e111. 10.1016/j.jand.2017.08.024 (2018). 29111090 10.1016/j.jand.2017.08.024

[9] Shan, Z. et al. Healthy eating patterns and risk of total and Cause-Specific mortality. *JAMA Intern. Med.* **183**, 142–153. 10.1001/jamainternmed.2022.6117 (2023). 36622660 10.1001/jamainternmed.2022.6117 PMC9857813

[10] Orlich, M. J. et al. Vegetarian dietary patterns and mortality in adventist health study 2. *JAMA Intern. Med.* **173**, 1230–1238. 10.1001/jamainternmed.2013.6473 (2013). 23836264 10.1001/jamainternmed.2013.6473 PMC4191896

[11] Sinha, R., Cross, A. J., Graubard, B. I., Leitzmann, M. F. & Schatzkin, A. Meat intake and mortality: a prospective study of over half a million people. *Arch. Intern. Med.* **169**, 562–571. 10.1001/archinternmed.2009.6 (2009). 19307518 10.1001/archinternmed.2009.6 PMC2803089

[12] Pan, A. et al. Red meat consumption and mortality: results from 2 prospective cohort studies. *Arch. Intern. Med.* **172**, 555–563. 10.1001/archinternmed.2011.2287 (2012). 22412075 10.1001/archinternmed.2011.2287 PMC3712342

[13] Storz, M. A., Ronco, A. L. & Hannibal, L. Observational and clinical evidence that plant-based nutrition reduces dietary acid load. *J. Nutr. Sci.* **11**, e93. 10.1017/jns.2022.93 (2022). 36405093 10.1017/jns.2022.93 PMC9641522

[14] Frassetto, L., Morris, R. C. Jr., Sellmeyer, D. E., Todd, K. & Sebastian, A. Diet, evolution and aging–the pathophysiologic effects of the post-agricultural inversion of the potassium-to-sodium and base-to-chloride ratios in the human diet. *Eur. J. Nutr.* **40**, 200–213. 10.1007/s394-001-8347-4 (2001). 11842945 10.1007/s394-001-8347-4

[15] Maurer, M., Riesen, W., Muser, J., Hulter, H. N. & Krapf, R. Neutralization of Western diet inhibits bone resorption independently of K intake and reduces cortisol secretion in humans. *Am. J. Physiol. Ren. Physiol.* **284**, F32–40. 10.1152/ajprenal.00212.2002 (2003). 10.1152/ajprenal.00212.2002 12388390

[16] Frassetto, L., Remer, T. & Banerjee, T. Dietary contributions to metabolic acidosis. *Adv. Chronic Kidney Dis.* **29**, 373–380. 10.1053/j.ackd.2022.03.008 (2022). 36175075 10.1053/j.ackd.2022.03.008

[17] Remer, T. & Manz, F. Potential renal acid load of foods and its influence on urine pH. *J. Am. Diet. Assoc.* **95**, 791–797. 10.1016/s0002-8223(95)00219-7 (1995). 7797810 10.1016/S0002-8223(95)00219-7

[18] Frassetto, L. A., Todd, K. M., Morris, R. C. Jr. & Sebastian, A. Estimation of net endogenous noncarbonic acid production in humans from diet potassium and protein contents. *Am. J. Clin. Nutr.* **68**, 576–583. 10.1093/ajcn/68.3.576 (1998). 9734733 10.1093/ajcn/68.3.576

[19] Ostrowska, J. & Janiszewska, J. Szostak-Węgierek, D. Dietary acid load and cardiometabolic risk Factors-A narrative review. *Nutrients* **12**. 10.3390/nu12113419 (2020). 10.3390/nu12113419 PMC7695144 33171835

[20] Han, E. et al. Association between dietary acid load and the risk of cardiovascular disease: nationwide surveys (KNHANES 2008–2011). *Cardiovasc. Diabetol.* **15**, 122. 10.1186/s12933-016-0436-z (2016). 27565571 10.1186/s12933-016-0436-z PMC5002186

[21] Fagherazzi, G. et al. Dietary acid load and risk of type 2 diabetes: the E3N-EPIC cohort study. *Diabetologia* **57**, 313–320. 10.1007/s00125-013-3100-0 (2014). 24232975 10.1007/s00125-013-3100-0

[22] Kiefte-de Jong, J. C. et al. Diet-dependent acid load and type 2 diabetes: pooled results from three prospective cohort studies. *Diabetologia* **60**, 270–279. 10.1007/s00125-016-4153-7 (2017). 27858141 10.1007/s00125-016-4153-7 PMC5831375

[23] Zhang, L., Curhan, G. C. & Forman, J. P. Diet-dependent net acid load and risk of incident hypertension in united States women. *Hypertension* **54**, 751–755. 10.1161/hypertensionaha.109.135582 (2009). 19667248 10.1161/HYPERTENSIONAHA.109.135582 PMC2777672

[24] Parohan, M. et al. Dietary acid load and risk of hypertension: A systematic review and dose-response meta-analysis of observational studies. *Nutr. Metab. Cardiovasc. Dis.* **29**, 665–675. 10.1016/j.numecd.2019.03.009 (2019). 31153745 10.1016/j.numecd.2019.03.009

[25] Arisawa, K. et al. Association of Dietary Acid Load with the Prevalence of Metabolic Syndrome among Participants in Baseline Survey of the Japan Multi-Institutional Collaborative Cohort Study. *Nutrients* **12**. 10.3390/nu12061605 (2020). 10.3390/nu12061605 PMC7352218 32486113

[26] Xu, H. et al. Modest U-Shaped association between dietary acid load and risk of All-Cause and cardiovascular mortality in adults. *J. Nutr.* **146**, 1580–1585. 10.3945/jn.116.231019 (2016). 27385761 10.3945/jn.116.231019

[27] Akter, S. et al. Dietary acid load and mortality among Japanese men and women: the Japan public health Center-based prospective study. *Am. J. Clin. Nutr.* **106**, 146–154. 10.3945/ajcn.117.152876 (2017). 28539378 10.3945/ajcn.117.152876

[28] Hejazi, E. et al. Dietary acid load and mortality from all causes, CVD and cancer: results from the Golestan cohort study. *Br. J. Nutr.* 1–7. 10.1017/s0007114521003135 (2021). 10.1017/S0007114521003135 34392847

[29] Hamajima, N. The Japan Multi-Institutional collaborative cohort study (J-MICC study) to detect gene-environment interactions for cancer. *Asian Pac. J. Cancer Prev.* **8**, 317–323 (2007). 17696755

[30] Takeuchi, K. et al. Study profile of the Japan Multi-Institutional Collaborative Cohort (J-MICC) study. *J. Epidemiol.* **31**, 660–668. 10.2188/jea.JE20200147 (2021). 32963210 10.2188/jea.JE20200147 PMC8593573

[31] Wakai, K. et al. Profile of participants and genotype distributions of 108 polymorphisms in a cross-sectional study of associations of genotypes with lifestyle and clinical factors: a project in the Japan Multi-Institutional Collaborative Cohort (J-MICC) study. *J. Epidemiol.* **21**, 223–235. 10.2188/jea.je20100139 (2011). 21467728 10.2188/jea.JE20100139 PMC3899413

[32] Craig, C. L. et al. International physical activity questionnaire: 12-country reliability and validity. *Med. Sci. Sports Exerc.* **35**, 1381–1395. 10.1249/01.Mss.0000078924.61453.Fb (2003). 12900694 10.1249/01.MSS.0000078924.61453.FB

[33] Tokudome, Y. et al. Relative validity of a short food frequency questionnaire for assessing nutrient intake versus three-day weighed diet records in middle-aged Japanese. *J. Epidemiol.* **15**, 135–145. 10.2188/jea.15.135 (2005). 16141632 10.2188/jea.15.135 PMC7851066

[34] Imaeda, N. et al. Reproducibility of a short food frequency questionnaire for Japanese general population. *J. Epidemiol.* **17**, 100–107. 10.2188/jea.17.100 (2007). 17545697 10.2188/jea.17.100 PMC7058456

[35] Kenneth, D. et al. Division of Vital Statistics. Deaths: Final Data for 2014 (2016). 27378572

[36] Aune, D. P. & Foods Antioxidant Biomarkers, and the risk of cardiovascular Disease, Cancer, and mortality: A review of the evidence. *Adv. Nutr.* **10**, S404–s421. 10.1093/advances/nmz042 (2019). 31728499 10.1093/advances/nmz042 PMC6855972

[37] Kurasawa, S. et al. Association of kidney function with cancer incidence and its influence on cancer risk of smoking: the Japan Multi-Institutional collaborative cohort study. *Int. J. Cancer*. **153**, 732–741. 10.1002/ijc.34554 (2023). 37158671 10.1002/ijc.34554

[38] Watanabe, T. et al. The significance of comprehensive metabolic phenotypes in cancer risk: A Japan Multi-Institutional collaborative cohort study. *Cancer Res. Commun.* **4**, 2986–2997. 10.1158/2767-9764.Crc-24-0249 (2024). 39470380 10.1158/2767-9764.CRC-24-0249 PMC11579844

[39] Murakami, K., Livingstone, M. B. E., Okubo, H. & Sasaki, S. Higher dietary acid load is weakly associated with higher adiposity measures and blood pressure in Japanese adults: the National health and nutrition survey. *Nutr. Res.* **44**, 67–75. 10.1016/j.nutres.2017.06.005 (2017). 28821319 10.1016/j.nutres.2017.06.005

[40] Abbasalizad Farhangi, M., Nikniaz, L. & Nikniaz, Z. Higher dietary acid load potentially increases serum triglyceride and obesity prevalence in adults: an updated systematic review and meta-analysis. *PLoS One*. **14**, e0216547. 10.1371/journal.pone.0216547 (2019). 31071141 10.1371/journal.pone.0216547 PMC6508739

[41] Adeva, M. M. & Souto, G. Diet-induced metabolic acidosis. *Clin. Nutr.* **30**, 416–421. 10.1016/j.clnu.2011.03.008 (2011). 21481501 10.1016/j.clnu.2011.03.008

[42] Farwell, W. R. & Taylor, E. N. Serum bicarbonate, anion gap and insulin resistance in the National health and nutrition examination survey. *Diabet. Med.* **25**, 798–804. 10.1111/j.1464-5491.2008.02471.x (2008). 18644066 10.1111/j.1464-5491.2008.02471.x

[43] Lloyd-Jones, D. M. et al. Prediction of lifetime risk for cardiovascular disease by risk factor burden at 50 years of age. *Circulation* **113**, 791–798. 10.1161/circulationaha.105.548206 (2006). 16461820 10.1161/CIRCULATIONAHA.105.548206

[44] Gami, A. S. et al. Metabolic syndrome and risk of incident cardiovascular events and death: a systematic review and meta-analysis of longitudinal studies. *J. Am. Coll. Cardiol.* **49**, 403–414. 10.1016/j.jacc.2006.09.032 (2007). 17258085 10.1016/j.jacc.2006.09.032

[45] Nguyen, T. V. et al. Associations of metabolic syndrome and metabolically unhealthy obesity with cancer mortality: the Japan Multi-Institutional collaborative cohort (J-MICC) study. *PLoS One*. **17**, e0269550. 10.1371/journal.pone.0269550 (2022). 35802721 10.1371/journal.pone.0269550 PMC9269937

[46] Boehme, A. K., Esenwa, C. & Elkind, M. S. Stroke risk Factors, Genetics, and prevention. *Circ. Res.* **120**, 472–495. 10.1161/circresaha.116.308398 (2017). 28154098 10.1161/CIRCRESAHA.116.308398 PMC5321635

[47] Blood pressure, cholesterol, and stroke in Eastern Asia. Eastern stroke and coronary heart disease collaborative research group. *Lancet* **352**, 1801–1807 (1998). 9851379

[48] Kita, Y. et al. Stroke incidence and case fatality in Shiga, Japan 1989–1993. *Int. J. Epidemiol.* **28**, 1059–1065. 10.1093/ije/28.6.1059 (1999). 10661648 10.1093/ije/28.6.1059

[49] Feigin, V. L., Lawes, C. M., Bennett, D. A., Barker-Collo, S. L. & Parag, V. Worldwide stroke incidence and early case fatality reported in 56 population-based studies: a systematic review. *Lancet Neurol.* **8**, 355–369. 10.1016/s1474-4422(09)70025-0 (2009). 19233729 10.1016/S1474-4422(09)70025-0

[50] Ikram, M. A., Wieberdink, R. G. & Koudstaal, P. J. International epidemiology of intracerebral hemorrhage. *Curr. Atheroscler Rep.* **14**, 300–306. 10.1007/s11883-012-0252-1 (2012). 22538431 10.1007/s11883-012-0252-1 PMC3388250

[51] Claassen, J. & Park, S. Spontaneous subarachnoid haemorrhage. *Lancet* **400**, 846–862. 10.1016/s0140-6736(22)00938-2 (2022). 35985353 10.1016/S0140-6736(22)00938-2 PMC9987649

[52] Mahmood, S. et al. Non-pharmacological management of hypertension: in the light of current research. *Ir. J. Med. Sci.* **188**, 437–452. 10.1007/s11845-018-1889-8 (2019). 30136222 10.1007/s11845-018-1889-8

[53] Steiger, S. et al. Sex dimorphism in kidney health and disease: mechanistic insights and clinical implication. *Kidney Int.* **107**, 51–67. 10.1016/j.kint.2024.08.038 (2025). 39477067 10.1016/j.kint.2024.08.038

[54] Iseki, K., Iseki, C., Ikemiya, Y. & Fukiyama, K. Risk of developing end-stage renal disease in a cohort of mass screening. *Kidney Int.* **49**, 800–805. 10.1038/ki.1996.111 (1996). 8648923 10.1038/ki.1996.111

[55] Jankie, S. & Pinto Pereira, L. M. Targeting insulin resistance with selected antidiabetic agents prevents menopausal associated central obesity, dysglycemia, and cardiometabolic risk. *Post. Reprod. Health*. **27**, 45–48. 10.1177/2053369120982753 (2021). 33356861 10.1177/2053369120982753

[56] Visniauskas, B. et al. Estrogen-mediated mechanisms in hypertension and other cardiovascular diseases. *J. Hum. Hypertens.* **37**, 609–618. 10.1038/s41371-022-00771-0 (2023). 36319856 10.1038/s41371-022-00771-0 PMC10919324

[57] Robinson, G. A. et al. Sex hormones drive changes in lipoprotein metabolism. *iScience* **24**, 103257. 10.1016/j.isci.2021.103257 (2021). 34761181 10.1016/j.isci.2021.103257 PMC8567005

[58] Nanri, A. et al. Dietary patterns and all-cause, cancer, and cardiovascular disease mortality in Japanese men and women: the Japan public health center-based prospective study. *PLoS One*. **12**, e0174848. 10.1371/journal.pone.0174848 (2017). 28445513 10.1371/journal.pone.0174848 PMC5405917

