# Supplementary figures and images for: Dietary acid load and mortality in the Japan Multi-Institutional Collaborative Cohort Study
Source: Sci Rep. 2025 Nov 21;15:41297. doi: 10.1038/s41598-025-25081-6 (PMC12639115; doi:10.1038/s41598-025-25081-6)

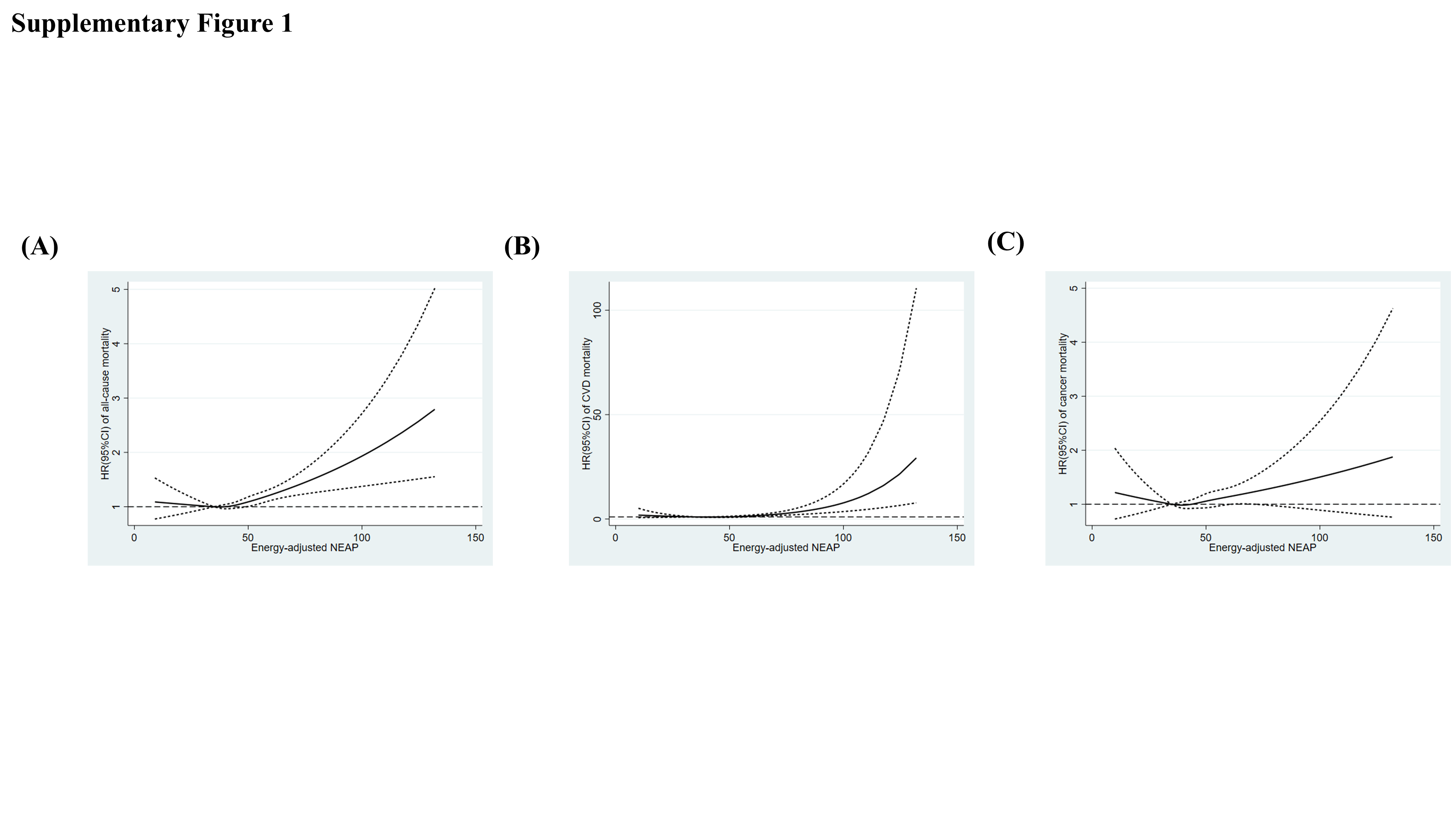

Supplement: Supplementary file 2 — Supplementary Material 2 [file 41598_2025_25081_MOESM2_ESM.tif]
