# Supplementary material for: Dietary acid load and mortality in the Japan Multi-Institutional Collaborative Cohort Study
Source: Sci Rep. 2025 Nov 21;15:41297. doi: 10.1038/s41598-025-25081-6 (PMC12639115; doi:10.1038/s41598-025-25081-6)
Supplement: Supplementary file 3 — Supplementary Material 3 [file 41598_2025_25081_MOESM3_ESM.docx]

**Figure legend of Supplementary Figure 1**

Supplementary Figure 1. HRs and 95% CIs for all-cause (A), cardiovascular (B), and cancer

(C) mortalities associated with NEAP by restricted cubic sprine analysis with number of knots

set to 4.
